# Spatio-Temporal Patterns of Major Bacterial Groups in Alpine Waters

**DOI:** 10.1371/journal.pone.0113524

**Published:** 2014-11-19

**Authors:** Remo Freimann, Helmut Bürgmann, Stuart E. G. Findlay, Christopher T. Robinson

**Affiliations:** 1 Institute of Molecular Health Sciences, Professorship of Genetics, ETH Zurich, Zurich, Switzerland; 2 Department of Aquatic Ecology, Swiss Federal Institute of Aquatic Science and Technology, Eawag, Dübendorf, Switzerland and Institute of Integrative Biology, ETH-Zurich, Zurich, Switzerland; 3 Department of Surface Waters – Research and Management, Swiss Federal Institute of Aquatic Science and Technology, Eawag, Kastanienbaum, Switzerland; 4 Cary Institute of Ecosystem Studies, Millbrook, New York, United States of America; Argonne National Laboratory, United States of America

## Abstract

Glacial alpine landscapes are undergoing rapid transformation due to changes in climate. The loss of glacial ice mass has directly influenced hydrologic characteristics of alpine floodplains. Consequently, hyporheic sediment conditions are likely to change in the future as surface waters fed by glacial water (kryal) become groundwater dominated (krenal). Such environmental shifts may subsequently change bacterial community structure and thus potential ecosystem functioning. We quantitatively investigated the structure of major bacterial groups in glacial and groundwater-fed streams in three alpine floodplains during different hydrologic periods. Our results show the importance of several physico-chemical variables that reflect local geological characteristics as well as water source in structuring bacterial groups. For instance, *Alpha*-, *Betaproteobacteria* and *Cytophaga-Flavobacteria* were influenced by pH, conductivity and temperature as well as by inorganic and organic carbon compounds, whereas phosphorous compounds and nitrate showed specific influence on single bacterial groups. These results can be used to predict future bacterial group shifts, and potential ecosystem functioning, in alpine landscapes under environmental transformation.

## Introduction

Heterotrophic bacteria are key players in the functional ecology of aquatic ecosystems, alpine waters in particular [1]. Their high metabolic capacity, phylogenetic variation and abundance enable bacterial assemblages to process and retain nutrients and chemical compounds under varying environmental conditions. Bacteria also represent an integral component within trophic food webs and global carbon cycling [2]. Glaciated alpine floodplains are an important source of fresh water as they are regions of high precipitation that is stored as snow/ice in winter and released during warm periods. Alpine environments not only modulate flow patterns and affect water chemistry but also provide microbe-mediated ecosystem services to waters used intensely by humans at lower elevations [3], [4]. Furthermore, alpine headwaters can have a high variability in species diversity within relative small geographical distance and thus contribute to the maintenance of microbial and functional diversity in the fluvial network [5], [6].

Members of bacterial groups are associated with metabolic traits and occupy habitat niches depending on the physico-chemical environment and apparent bacterial (single cell) functional plasticity [7]. Glaciated alpine floodplains comprise a mosaic of groundwater-fed (krenal) and glacial (kryal) streams differing in physico-chemical characteristics and thus different habitat niches [8]. The hyporheic zone of streams, in particular, is known as a biological hotspot of microbial abundance and functioning within these landscapes [9]. Environmentally-induced recession of glaciers and current shifts in precipitation patterns will affect alpine waters and associated habitats by reducing kryal systems at the landscape scale [10], [11]. This reduction includes shifts in quality, quantity and timing of glacially-released organic matter and nutrients as well as changes in flow regimes that can alter hyporheic sediment characteristics [12]. Thus, ecological shifts in bacterial structure in conjunction with potential functioning are likely to occur. The underlying mechanics are dependent on the magnitude and rate of environmental change and are at least partially determined by the properties of the contemporary bacterial assemblage [13]. The magnitude of ecological change will ultimately depend on the degree of functional redundancy/plasticity and is likely manifest in a combination of changes involving cell abundances, single cell metabolic activities and shifts in bacterial composition [7].

In previous studies, we documented a strong linkage between bacterial structure and function in hyporheic sediments [6]. We showed how ecological strategies of communities (generalists vs. specialists) influence the trajectory of community changes due to altered physico-chemical properties [13]. However, these earlier results only focused on the structure of bacterial assemblages without taking into account phylogenetic identities. In the present study, we quantitatively examined the phylogeny of major freshwater bacterial groups present in hyporheic sediments via catalyzed reporter deposition fluorescence in-situ hybridization (CARD-FISH) within three alpine glacial catchments. The catchments differed in the degree of deglaciation and thus covered a wide range of habitat types within krenal and kryal systems. These different physic-chemical conditions were associated with differences in bacterial groups and the broad range in habitat and bacterial attributes probably encompass future scenarios for alpine landscapes under transformation.

## Material and Methods

**Figure 1:**
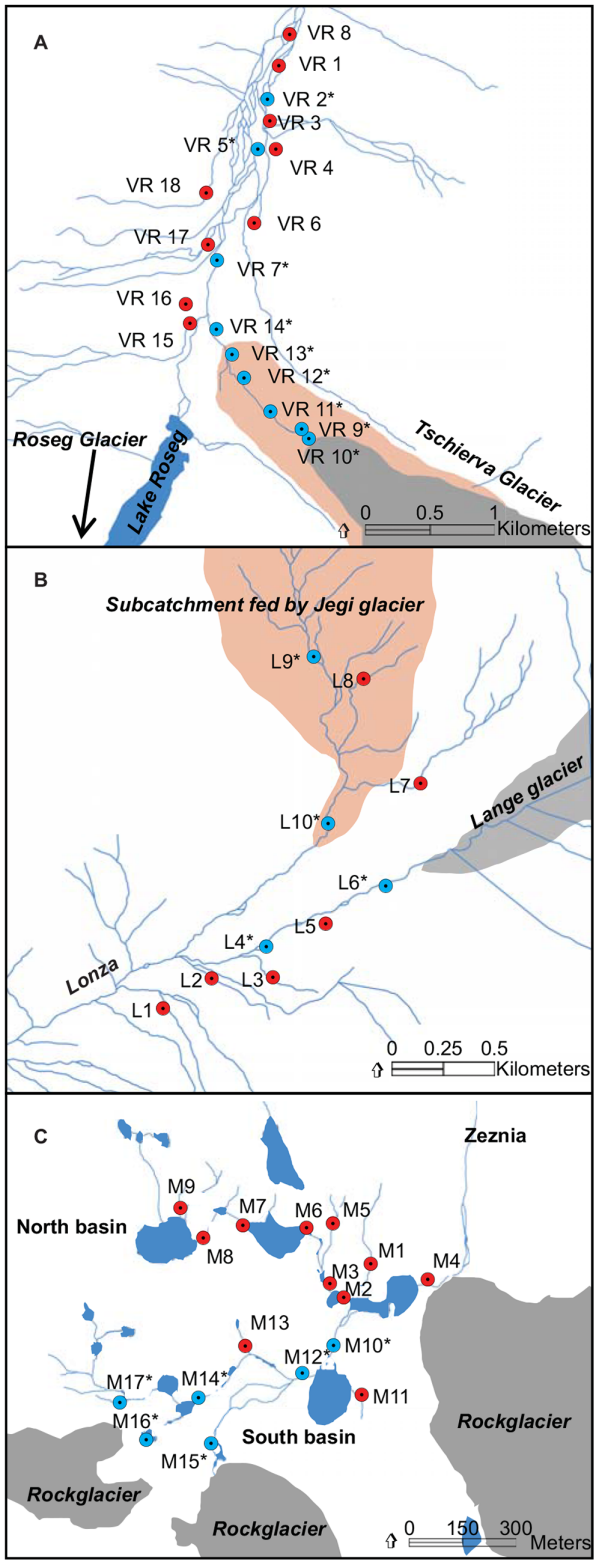
Map of the study sites in the three catchments, A: Val Roseg (VR), B: Loetschental (L) and C: Macun (M). Kryal sites are depicted in blue and annotated with an asterisk. Glaciers and the moraine area in VR and the sub-catchment in L are depicted in light grey and orange, respectively.

We collected hyporheic sediments (∼10 to 20 cm depth) from 45 stream sites in three alpine floodplains, Val Roseg (VR, 9°53′53′′E, 46°29′24′′N), Loetschental (L, 07°49'03''E, 46°25'08''N) and Macun (M, 10°07'31''E, 46°43'51''N), that differed in their degree of deglaciation (% of the catchment area covered by ice, data provided by the Federal Office for Environment and the Swiss National Park) and general landscape features such as the presence of interconnected lakes (Table 1, Figure 1). Sampling sites along streams were categorized into krenal and kryal systems, depending on the connectivity to glaciers/glacial meltwater and based on water chemistry patterns (Figure 1) [6]. Macun had a perennially-reduced glacial water input, and more specific characterizations of these catchments can be found elsewhere [6]. Sites were sampled during three distinct hydrological periods: glacial ablation in summer (August: A), winter stagnation (October: O) and snowmelt input in spring (June: J) in Val Roseg and Loetschental and for A and O in Macun. No specific permission was required for the sampling in VR and L. Permission for the sampling campaign in M was issued by the Swiss National Park. The study did not involve endangered or protected species.

**Table 1:** Basic characteristics of the three catchments.

Catchment	Val Roseg	Loetschental	Macun
Coordinates	9°53′53′′E, 46°29′24′′N	07°49'03''E, 46°25'08''N	10°07'31''E, 46°43'51''N
Altitude [m a.s.l.]	1766–4049	1375–3200	2616–3046
Catchment area [km^2^], (% glaciated)	66.5 (30.1)	77.8 (36.5)	3.6 (18.8)*
Annual precipitation [m]	1.6	1.1	0.9
Mean discharge [m^3^ s^−1^]	28.5	37.2	ND
Mean water temperature of main channel [°C] (range)	3.6 (1–12)	4 (0.1–10.9)	2.9 (0.1–19.2)
Interconnected lakes	No	No	Yes
Geology, dominating minerals	Crystalline bedrock, diorite, granite	Crystalline bedrock, amphibolite, gneiss	Crystalline bedrock, ortho-gneiss

Abbreviation: ND, no data; *rock glaciers.

A 0.5 ml aliquot of collected sediment was suspended in 1.11 ml paraformaldehyde (2%, final concentration) in an Eppendorf tube and fixed for 24 h at 4°C followed by three washing steps with 1× PBS and 5 min centrifugation at 10,000 g between washing steps. Samples were then stored at −20°C in a 1∶1 mix of PBS/ethanol until further processing [14]. Cell detachment was done by sonication (Branson Digital Sonifier 250, Danbury, USA, 5-mm tapered microtip, actual output of 20 W, 30 s). Catalyzed reporter deposition fluorescence in-situ hybridization (CARD-FISH) was performed following the protocol of Pernthaler *et al*. [15] paired with a high throughput imaging system [16]. Horseradish labeled FISH probes (Biomers Inc, Ulm) EUB I-III targeting the domain *Bacteria* (*EUBI-III*) [17], Alf968 and Bet42a affiliated with classes of *Alphaproteobacteria* (*Alph*) and *Betaproteobacteria* (*Bet*) of the phylum *Proteobacteria*, respectively [18], [19], and CF319a assigned to the class of *Cytophaga-Flavobacteria* (*CF*) within the phylum *Bacteroidetes* [20] were used to quantify microbial groups within the stream sediments. The domains and classes are expressed as percentage of total bacterial abundance as assessed by counterstaining bacterial cells with 4′,6-diamidino-2-phenylindole (DAPI) (Sigma-Aldrich Co) [21].

Specific conductance (µS cm^−1^ at 20°C) and temperature were measured in the field with a conductivity meter (LF323, WTW, Weilheim, Germany). Surface water samples were analyzed for dissolved organic carbon (DOC), particulate organic carbon (POC), total inorganic carbon (TIC), ammonium (NH_4_-N), nitrite (NO_2_-N), nitrate (NO_3_-N), dissolved organic nitrogen (DON), particulate organic nitrogen (PN), phosphate (PO_4_-P), dissolved phosphorus (DP) and particulate phosphorus (PP) according to standard protocols detailed in Tockner *et al*. [22]. Sediments were analyzed for pH [23] and organic matter content (OM) as ash free dry mass. Grain size distribution of sediment was assessed by sieving with mesh sizes of 6.3, 2.0, 1.0, 0.5, 0.25, 0.125 and 0.063 mm. The D90/D10 gradation index was then calculated using GRADISTAT software [24]. The raw physico-chemical data have been published elsewhere [6].

To assess the explained variance of physico-chemical variables on CARD-FISH based bacterial community composition, we performed a redundancy analysis (RDA) based on forward selected environmental variables [25]. Significance of physico-chemical constraining variables and constrained RDA axes were tested by permutation tests (999 permutations) [26]. The relative contribution of physico-chemical variables to the constraint variation of single RDA axis was assessed via canonical correlation coefficients. Factor (catchment, water source and season) and vector (i.e. physico-chemical variables) fitting was performed on the first two RDA axes to assess their significance and relationship (r^2^) to the phylogenetic patterns shown on these two axes. We tested all levels of interactions for the factor fitting, i.e. the influence of a single factor and all double and triple interactions of the respective factors. Pearson's product moment correlation was used to correlate physico-chemical variables with single bacterial groups and correlations were tested using Algorithm AS 89 [27]. Lastly, comparison of hybridization rates between water source, seasons and catchments were done using ANOVAs followed by Tukey's honest significance text (Tukey's HSD). Analyses were based on 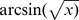 transformed percentage values of CARD-FISH results and log +1 transformed physico-chemical variables. All analysis were done using the R statistical environment [28].

## Results

**Figure 2:**
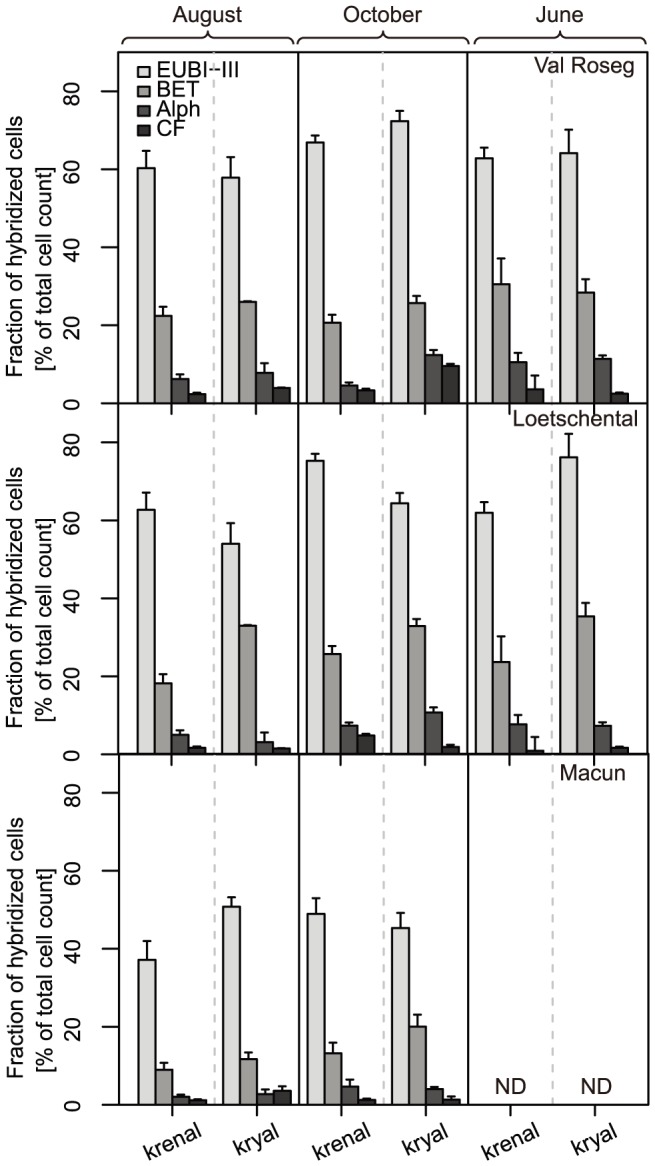
Bar plots (+1SE) of the relative abundance of major bacterial groups in the Val Roseg (A), Loetschental (B) and Macun (C) catchment. Bar groups are split within a panel by season and water source. ND: No data.

**Figure 3:**
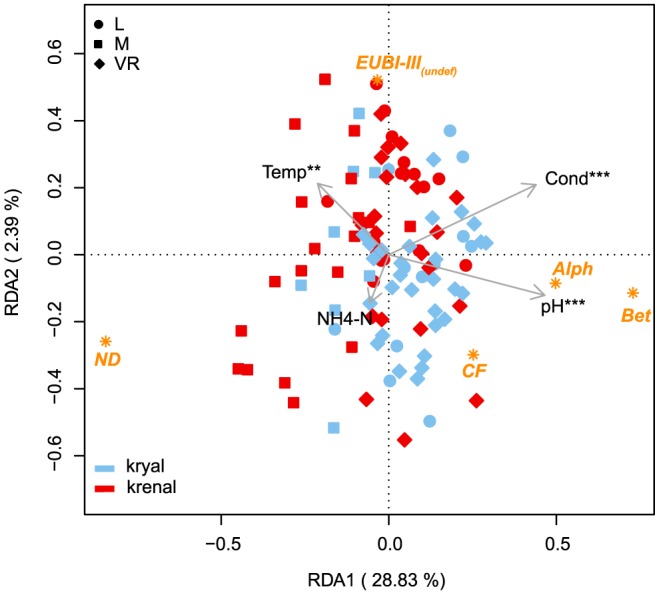
Biplot of the redundancy analysis based on the CARD-FISH data. Red symbols correspond to krenal sampling sites, whereas blue symbols depict kryal sites. Centroids of the respective probes are given: beta-*proteobacteria* (*Bet*), alpha-*proteobacteria* (*Alph*), Cytophaga-Flavobacteria (*CF*), Eubacteria excluding *Alph*, *Bet* and *CF* (*EUBI-III_(undef)_*) and not-defined DAPI positive cells (ND). Arrows depict the forward selected physico-chemical variables. Asterisks depict significantly tested variables (* p<0.05, ** p<0.01). Explained variation for the first two constraint axes is given.

Sediments from L and VR had a higher mean *EUBI-III* hybridization rate than M (Figure 2, Table 2, Tukey's HSD: P<0.001). *EUBI-III hybridization rate* in Macun showed proportionally the smallest overlap with the further defined lower taxonomic level (classes, Figure 2). We refer to the not further defined *Bacteria* group (i.e., %EUBI-III – (%Alph + %Bet + %CF)) as *EUBI-III_(undef)_* (Figure 3). There was a higher *EUBI-III* hybridization rate in winter compared to summer (Tukey's HSD: P<0.01).

**Table 2:** ANOVA results (F values) for bacterial group abundance.

F-statistic parameter	Catchment (C)	Sampling date (S)	Water source (W)	C×S	C×W	S×W	C×S×W
*EUBI-III*	7.49***	6.68**	4.55*	1.19	3.26*	3.89*	3.1*
	VR	4.68*	0.4	-	-	0.85	-
	L	4.14*	0.11	-	-	5.08*	-
	M	0.16	0.49	-	-	1.64	-
*BET*	20.22***	1.54	5.78*	1.39	4.18*	0.79	3.1
	VR	3.49*	1.02	-	-	1.06	-
	L	0.67	12.84**	-	-	0.48	-
	M	5.44*	3.14	-	-	0.59	-
*Alph*	5.57**	5.98**	4.25*	1.06	1.23	1.51	1.05
	VR	2.73	5.79*	-	-	2.3	-
	L	6.48**	0.11	-	-	1.65	-
	M	3.35	0	-	-	0.37	-
*CF*	8.26***	3.18*	5.37*	4.18**	4.68*	0.02	7.38***
	VR	10.31***	10.89**	-	-	9.61***	-
	L	1.87	0.76	-	-	1.4	-
	M	3.13	4.04	-	-	3.45	-

Catchment (C), Sampling date (S) and Water source (W) were used as independent variables.

*Bet* was the most abundant group according to CARD-FISH and was lowest in M compared to the other two catchments (Tukey's HSD: p<0.001). *Bet* had on average highest abundance in kryal sediments in L (Tukey's HSD: P<0.001). *Alph* showed highest abundance in kryal sediments in VR (Tukey's HSD: p<0.05) and higher abundance in winter compared to spring in L (Tukey's HSD: p<0.01). *CF* was least abundant and VR had higher *CF* abundance compared to the other two catchments (Tukey's HSD: p<0.001) and showed a peak in kryal sediments in winter (Tukey's HSD: p<0.001). See also table 2 for detailed results of ANOVAs.

**Figure 4:**
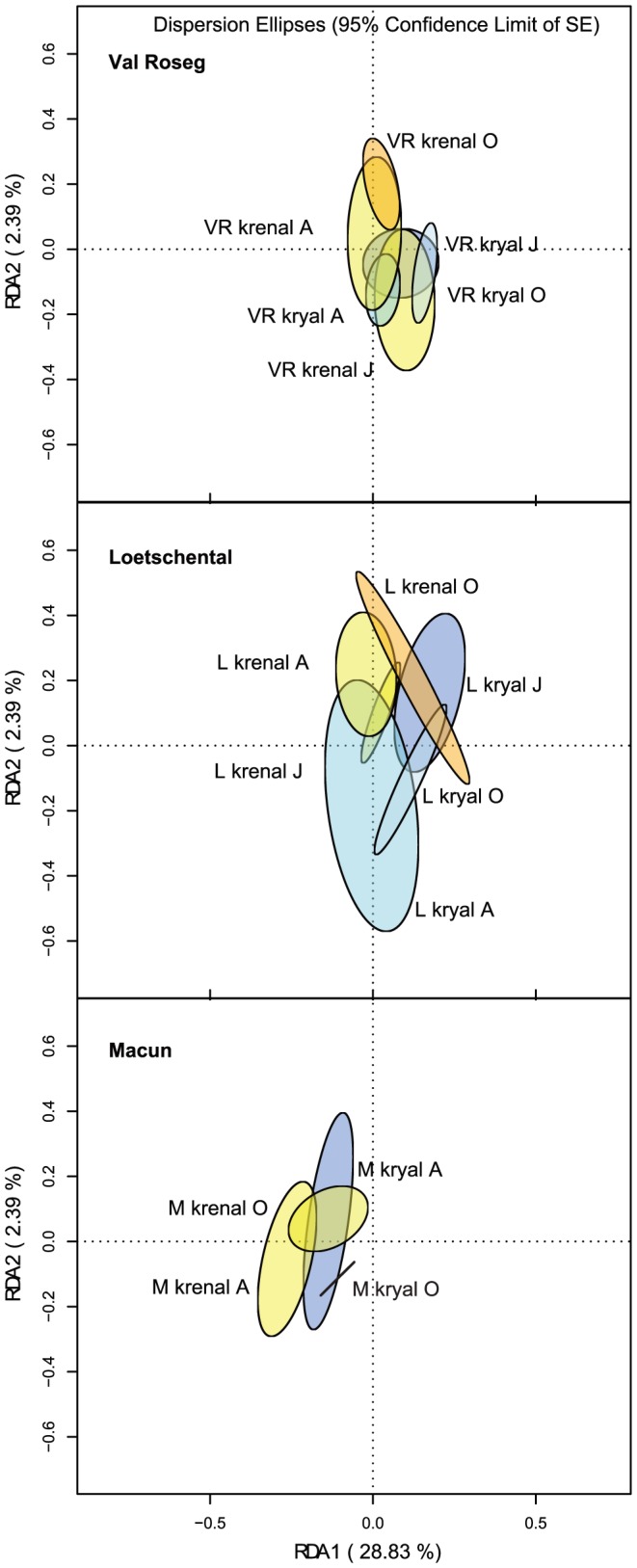
Dispersion ellipses fitted on the biplot of the redundancy analysis based on the CARD-FISH data constraint by physico-chemical variables (**Figure 3**). Dispersion ellipses split by catchments for different water sources and seasons are shown and depict the standard error of weighted average scores (confidence limit  = 0.95).

**Figure 5:**
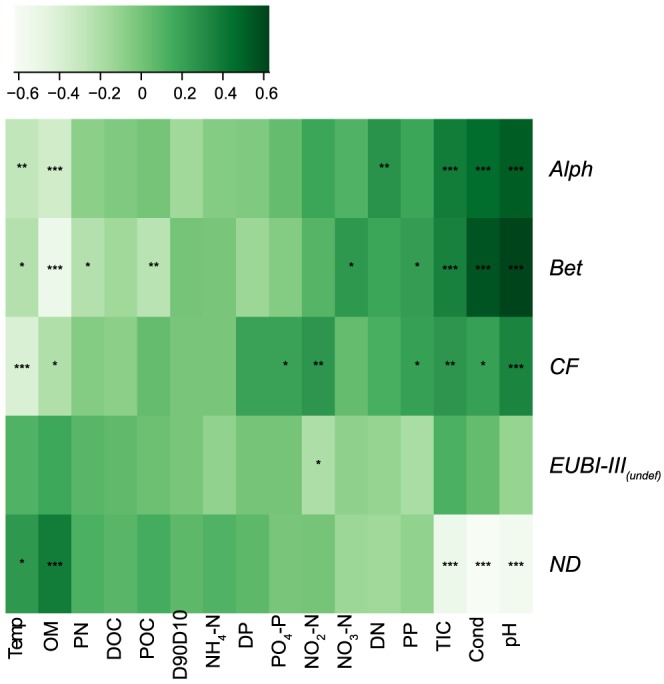
Heat-map of the Pearson correlations of beta-*proteobacteria* (*Bet*), alpha-*proteobacteria* (*Alph*), Cytophaga-Flavobacteria (*CF*), Eubacteria excluding *Alph*, *Bet* and *CF* (*EUBI-III_(undef)_*) and not-defined DAPI positive cells (ND) to physico-chemical variables. Asterisk indicate the p-value (*: p<0.05; **: p<0.01; ***: p<0.001) of correlations between paired samples.

RDA based on forward selected environmental variables explained 31.9% of the total variation and revealed a differentiation of the three catchments concerning their phylogenetic group structuring (Figure 3). Catchment and water source were significantly (p<0.05) fitted on the RDA biplot (r^2^ = 0.09 and 0.05, respectively). This was also true for the interaction term of catchment, sampling date and water source (P<0.001, r^2^ = 0.30). The ordination ellipses in figure 4 depict the standard error of weighted average scores (confidence limit  = 0.95) of the interaction sampling date × water source split by catchments. This figure is based on the RDA biplot of figure 3. Significant differences between water sources within a specific season can be expected when the respective ellipses do not overlap. The pH, conductivity, temperature and NH_4_-N contributed 23.2%, 2.1%, 1.7% and 1.8% to the total 28.8% explained variation on the first RDA axis (Figure 3). The pH, conductivity and temperature could also be fitted a-posteriori as independent variables on the biplot (r^2^ = 0.53, 0.46 and 0.13, respectively, p<0.01). NH_4_-N could not be significantly fitted on the RDA (p = 0.73) (Figure 3). Additionally, we fitted all non-forward selected environmental variables on the first two RDA axes of the forward selected model to assess their relative importance in structuring the bacterial communities (Table S1). OM, TIC and PP showed significant a-posteriori fitting on the first two RDA axes (p<0.01). The abundances of the different bacterial groups could be linked to several physico-chemical parameters (Figure 5). All bacterial groups were negatively correlated with OM and temperature, and *Bet* also was negatively correlated with POC and PN. TIC, Cond and pH were positively correlated with *Alph*, *Bet* and *CF*. PP was positively correlated with *Bet* and *CF*, whereas *Alph* was correlated with DN. PO_4_-P and NO_2_-N showed a positive Pearson correlation with *CF*, whereas *Bet* was correlated with NO_3_-N.

## Discussion

Our results showed that bacterial group composition in hyporheic sediments of streams in glaciated alpine floodplains have a strong spatio-temporal dynamic. Physico-chemical differences between catchments, such as pH, conductivity and temperature, were related to geological and geographical characteristics, and dictate the coarse-scale boundary conditions on bacterial group composition. These factors have been described previously to drive assemblage composition and diversity in soils as well as in stream sediments, although not at the group level assessed here [6], [29], [30]. Importantly, these previously documented variables affecting bacterial structural patterns at a higher phylogenetic level (i.e. automated ribosomal intergenic spacer analysis) within these catchments were largely congruent to the variables structuring the lower phylogenetic level structures (i.e. CARD-FISH) in this study [13]. This congruence was also true for drivers (i.e. OM, POC, PP, TIC and NO_2_-N) linked more specifically to single bacterial groups (see Figure 5). Furthermore, enzymatic activities measured in the above mentioned study were used as constraints variables in an RDA analysis based on the CARD-FISH data and revealed significant correlations to the phylogenetic group structuring (29.6% total explained variation, see Figure S1). Results from the automated ribosomal intergenic spacer analysis and the enzyme activities were also fitted a-posteriori on the RDA biplot produced here (Figure S2). Taken together, these results underpin the separation of the different catchments based on the CARD-FISH data and indicate a substantial coupling of bacterial groups and their potential metabolic capabilities.

Most of the examined variables differed between the two water sources (kryal vs krenal) [6] and partly induced the observed seasonal turnover in assemblage structure (Figure 4). Kryal systems are rich in PP during summer ablation and favored *Bet* and *CF* within kryal habitats in VR and L (Figure 5) [22]. Catchment M showed reduced abundance of *Bet*, which was correlated to reduced PP input into kryal streams here [6]. Also, NH_4_-N and NO_2_-N levels are high in summer in kryal waters with the latter being positively correlated with the abundance of *Alph*, *Bet* and *CF* [13]. During winter, kryal systems become physico-chemically more equal to krenal systems as glacial water input diminishes. Nevertheless, there was no distinct shift in kryal group composition towards a krenal one at the floodplain scale within the three catchments, suggesting relatively resistant local bacterial assemblages despite the aforementioned coupling of structure and function (see dispersion ellipses in figure 4). Experiments where kryal and krenal bacterial communities were cross-transplanted between their natural habitats revealed a high structural resistance along with a pronounced functional plasticity in response to physico-chemical disturbance [13]. Nevertheless, at this higher phylogenetic level and at the landscape scale, there was a difference in coupling of bacterial structure and functions within krenal and kryal systems with the latter having higher congruency [6]. At the phylogenetic level studied here, it seems that functional shifts, which depend on apparent redundancy/plasticity, are linked to structural changes of similar extent in both water systems. This discrepancy is likely due to non-detected seasonal changes of bacterial species within bacterial groups. That is, kryal systems can be seen as dominated by specialists whereas krenal systems tend to harbor generalists (on the species level) [6]. Thus, kryal community composition changes in response to new functional requirements, since the level of plasticity/redundancy in the community is low. Krenal communities, on the other hand, don't shift as strongly, since the communities plasticity/redundancy is high. Such differences in community shifts between the systems cannot be detected at the level of bacterial groups, if the community shift occurs mainly within and not across groups. Regardless, the coupling of bacterial groups to functions becomes evident when comparing different catchments.

A dominance of *Proteobacteria* has been shown before in snow-packs as well as in different glacial habitats [31], [32]. Thus, the low total abundance of *Proteobacteria* in catchment M may be due to an interactive effect of decreased glacial water input and a generally harsh environment due to the high altitude (Table 1). *Bet* and partly *Alph* are often predominant in sediments in lower elevation streams [33], [34]. *Bet* has been described as a diverse group dominating freshwater systems of different oligotrophic states [35], [36] and are highly competitive at the initial state of biofilm development [37]. This trait may be the reason that they can compete well in kryal sediments in VR and L, as these habitats experience mechanic abrasion induced by flow as well as low OM. *Bet* also have been shown to be involved in the degradation of pollutants, and thus may provide beneficial ecosystem functions within alpine floodplains. Indeed, precipitation driven pollutant inputs may favor *Bet* as proposed by Brümmer et al. [35]. Biofilm bacterial assemblages in urban rivers have been shown to be dominated by *Bet* and *CF*, which may be linked to their pollution load [38]. Similar to our findings in VR, Araya *et al*. also found a peak of *CF* during the winter season in urban rivers which can be linked to lower temperatures (Figure 5) [8], [38].

In summary, ongoing (and rapid) glacial recession and shift in water source (i.e. physico-chemical habitat template) will likely influence bacterial group composition in glaciated alpine floodplains. Differences between kryal and krenal systems were not as distinct at this taxonomic resolution (Figure 3, Figure S2) such that a dramatic change in bacterial group diversity at the landscape scale is expected. At a longer time scale, there will be other environmental changes concomitant to the glacial mass loss induced physico-chemical habitat shifts, such as changes in terrestrial vegetation, increasing OM inputs and increased water temperatures. These changes will likely induce subtle shifts in major bacterial groups towards a krenal assemblage composition and thus a homogenization of bacterial assemblages. Ultimately, alpine bacterial assemblage structure may become more similar to present lower elevation stream bacterial assemblages, thereby affecting ecosystem functioning and services.

## Supporting Information

Figure S1 **Biplot of the redundancy analysis based on the CARD-FISH data constrained by enzymatic activities measured in a previous study.** Dark grey dots correspond to krenal sampling sites, whereas light grey dots depict kryal sites. Centroids of the respective probes are given: Beta*proteobacteria* (*Bet*), Alpha*proteobacteria* (*Alph*), Cytophaga-Flavobacteria (*CF*), Eubacteria excluding *Alph*, *Bet* and *CF* (*EUBI-III_(undef)_*) and not-defined DAPI positive cells (ND). Arrows depict the forward selected Hellinger transformed enzyme activities (expressed as nmol m^2^ h^−1^). Asterisks depict significantly tested enzymes (* p<0.05, ** p<0.01). Dispersion ellipses for the catchments and different water sources are shown and depict the standard error of weighted average scores (confidence limit  = 0.95). Explained variation for the first two constraint axes is given.
(EPS)

Figure S2 **Biplot of the redundancy analysis based on the CARD-FISH data constrained by physico-chemical variables (not shown but equal to** **Figure 3** **).** Dark grey dots correspond to krenal sampling sites, whereas light grey dots depict kryal sites. Centroids of the respective probes are given: Beta*proteobacteria* (*Bet*), Alpha*proteobacteria* (*Alph*), Cytophaga-Flavobacteria (*CF*), Eubacteria excluding *Alph*, *Bet* and *CF* (*EUBI-III_(undef)_*) and not-defined DAPI positive cells (ND). Arrows depict the a posteriori fitted enzymatic activities (p<0.05) and operational taxonomic units (p<0.01) from a previous study. Dispersion ellipses for the catchments and different water sources are shown and depict the standard error of weighted average scores (confidence limit  = 0.95). Explained variation for the first two constraint axes is given.
(EPS)

Table S1 **Vector fitting of physico-chemical variables on the first two axis of the RDA-Biplot (** **Figure 3** **).**
(DOCX)
