# Supplementary material for: Spatio-Temporal Patterns of Major Bacterial Groups in Alpine Waters
Source: PLoS One. 2014 Nov 19;9(11):e113524. doi: 10.1371/journal.pone.0113524 (PMC4237416; doi:10.1371/journal.pone.0113524)
Supplement: Table S1 — Vector fitting of physico-chemical variables on the first two axis of the RDA-Biplot ( Figure 3 ). (DOCX) [file pone.0113524.s003.docx]

| **Table S1** Vector fitting of physico-chemical variables on the first two axis of the RDA-Biplot (Figure 3). | | |
| --- | --- | --- |
| Variable | r^2^ (RDA1, RDA2) | P-Value |
| DOC | 0.02 | 0.31 |
| POC | 0.04 | 0.13 |
| TIC | 0.28 | <0.001 |
| NH_4_-N | 0.01 | 0.73 |
| NO_2_-N | 0.06 | 0.05 |
| NO_3_-N | 0.00 | 0.91 |
| DN | 0.01 | 0.70 |
| PN | 0.05 | 0.11 |
| PO_4_-P | 0.00 | 0.96 |
| DP | 0.01 | 0.72 |
| PP | 0.07 | 0.02 |
| Cond | 0.46 | <0.001 |
| Temp | 0.13 | <0.001 |
| OM | 0.31 | <0.001 |
| pH | 0.53 | <0.001 |
| D90D10 | 0.01 | 0.73 |
